# Evaluation of Carbonic Anhydrase, Acetylcholinesterase, Butyrylcholinesterase, and α-Glycosidase Inhibition Effects and Antioxidant Activity of Baicalin Hydrate

**DOI:** 10.3390/life13112136

**Published:** 2023-10-29

**Authors:** Lokman Durmaz, Hasan Karagecili, İlhami Gulcin

**Affiliations:** 1 Department of Medical Services and Technology, Cayirli Vocational School, Erzincan Binali Yildirim University, Erzincan 24500, Türkiye; lokmandurmaz25@gmail.com; 2 Department of Nursing, Faculty of Health Sciences, Siirt University, Siirt 56100, Türkiye; hasankaragecili@siirt.edu.tr; 3 Department of Chemistry, Faculty of Science, Ataturk University, Erzurum 25240, Türkiye

**Keywords:** baicalin hydrate, acetylcholinesterase, antioxidant activity, α-glycosidase, butyrylcholinesterase, carbonic anhydrase, phenolic compounds

## Abstract

Baicalin is the foremost prevalent flavonoid found in *Scutellaria baicalensis*. It also frequently occurs in many multi-herbal preparations utilized in Eastern countries. The current research has assessed and compared the antioxidant, antidiabetic, anticholinergic, and antiglaucoma properties of baicalin hydrate. Baicalin hydrate was tested for its antioxidant capacity using a variety of techniques, including N,N-dimethyl-p-phenylenediamine dihydrochloride radical (DMPD^•+^) scavenging activity, 2,2′-azinobis-(3-ethylbenzothiazoline-6-sulphonate) radical (ABTS^•+^) scavenging activity, 1,1-diphenyl-2-picrylhydrazyl radical (DPPH^•^) scavenging activity, potassium ferric cyanide reduction ability, and cupric ions (Cu^2+^) reducing activities. Also, for comparative purposes, reference antioxidants, such as butylated hydroxyanisole (BHA), Trolox, α-Tocopherol, and butylated hydroxytoluene (BHT) were employed. Baicalin hydrate had an IC_50_ value of 13.40 μg/mL (r^2^: 0.9940) for DPPH radical scavenging, whereas BHA, BHT, Trolox, and α-Tocopherol had IC_50_ values of 10.10, 25.95, 7.059, and 11.31 μg/mL for DPPH^•^ scavenging, respectively. These findings showed that baicalin hydrate had comparably close and similar DPPH^•^ scavenging capability to BHA, α-tocopherol, and Trolox, but it performed better than BHT. Additionally, apart from these studies, baicalin hydrate was tested for its ability to inhibit a number of metabolic enzymes, including acetylcholinesterase (AChE), butyrylcholinesterase (BChE), carbonic anhydrase II (CA II), and α-glycosidase, which have been linked to several serious illnesses, such as Alzheimer’s disease (AD), glaucoma, and diabetes, where the K_i_ values of baicalin hydrate toward the aforementioned enzymes were 10.01 ± 2.86, 3.50 ± 0.68, 19.25 ± 1.79, and 26.98 ± 9.91 nM, respectively.

## 1. Introduction

**Figure 1:**
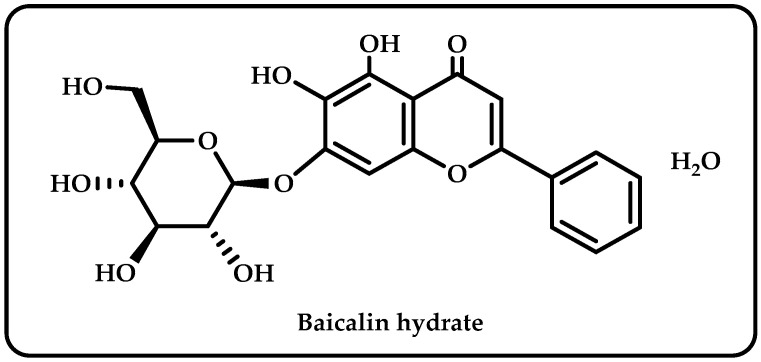
The molecule structure of baicalin hydrate.

*Scutellaria baicalensis* is a ubiquitous plant in the Labiatae family, found in large quantities in different parts of world [1]. Baicalin was an isolated flavonoid derived from the roots of *Scutellaria baicalensis*. It claims to have diverse pharmacological effects, including anti-inflammatory, antioxidant, antibacterial, antifungal, and anticancer effects [2]. It has also been utilized to treat hypertension, sleeplessness, inflammation, bleeding, and dysentery [3]. As seen in Figure 1, baicalin hydrate is a natural substance comprising four phenolic hydroxyl groups; it has antiviral, antibacterial, and anti-inflammatory properties [4].

It is known that there is a balance between oxidative stress and antioxidant defense mechanism. When the antioxidant capacities of endogenous antioxidants are exceeded by the formation of reactive oxygen species (ROS), a metabolic imbalance called oxidative stress occurs [5]. ROSs are formed in excess amounts during metabolism and can cause oxidative damage. They may oxidize and disrupt biomolecules such as lipids, proteins, and nucleic acids, which are the main building blocks of cell membranes and can cause cell death. Also, ROSs have been linked to the pathogenesis of many different human disorders that disrupt the operation of the genetic mechanism [6]. Nonetheless, antioxidants may postpone or reduce such oxidative damage, preventing and alleviating illnesses caused by oxidative stress [7]. Furthermore, antioxidants, which are biologically active substances that cannot be produced by the body, can strengthen the body’s antioxidant defenses when taken with food [8,9,10]. The natural sources of fruits and vegetables have a powerful and broad spectrum of antioxidants. In this context, antioxidants obtained from these sources have the ability to remove ROS easily. As a result, naturally occurring antioxidants of dependable plant-based origin are valued and sought as a complement to manufactured antioxidants with detrimental consequences [11].

Polyphenols, especially flavonoids, have been the subject of extensive research due to their abundance in plant-based materials [12]. The benefits of phenolics, which include antioxidant, anti-inflammatory, and anti-tumor activities, have been demonstrated in several papers [13]. Plants create secondary metabolites, such as phenolic compounds, to help them survive. These compounds have a variety of biological functions but are particularly good at acting as an antibiotic and an antioxidant [14]. A multitude of biological benefits, including antioxidant and free radical scavenging properties, have been linked to flavonoids and phenolic compounds that are extensively present in plants [15].

**Figure 2:**
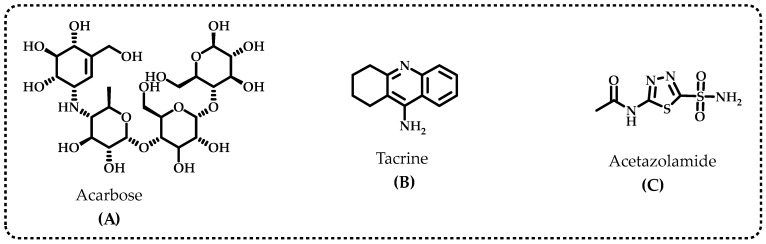
The standard inhibitors used for the α-glycosidase (**A**), acetylcholinesterase (AChE) and butyrylcholinesterase (BChE) (**B**) and human carbonic anhydrase II isoenzyme (hCA II) (**C**).

Diabetes mellitus (DM) is considered an epidemic as it is the fourth great contributing factor to mortality in developed countries. The etiology of type-2 DM (T2DM), which includes lifestyle choices, dyslipidemia, and genetic susceptibility, is complicated because of the number of variables involved [16]. Inhibition of α-glycosidase, one of the most significant digestive enzymes that catalyzes the breakdown of dietary polysaccharides, is a primary target for treatment of T2DM [17]. Miglitol, 1-deoxynojirimycin, voglibose, and acarbose are the most common α-glycosidase inhibitors (Figure 2A). α-Glycosidase inhibitors (AGIs) reduce postprandial plasma glucose levels and delay the absorption of monosaccharides from the small intestine. These characteristics may allow AGIs to be used to cure T2DM and obesity [18]. The improvement of postprandial glucose levels, fasting blood glucose levels, acute insulin secretion, and insulin sensitivity are just a few of the ways polyphenols may affect the metabolism of carbohydrates. Limiting the pace at which glucose enters circulation after leaving the intestines is a method for preventing DM [11]. Acarbose, miglitol, and voglibose are the currently prescribed medications that prevent the absorption of glucose; however, they have undesirable side effects since they are not targeted medications [19]. Various natural polyphenols have been shown to have anti-AD properties and α-glycosidase inhibition profiles. These findings indicate that compounds obtained from natural sources have importance in the treatment of AD and T2DM [20].

The most frequent cause of dementia is Alzheimer’s disease (AD), and an excessive buildup of the amyloid-β peptide in the brain is a crucial pathological alteration in AD. Furthermore, memory loss and severe impairment in cognitive function are the key clinical indicators of this disease [21]. The body contains significant quantities of acetylcholinesterase (AChE), a crucial enzyme that quickly hydrolyzes acetylcholine, a neurotransmitter that exists in the synaptic gap, to stop nerve impulses at cholinergic synapses while maintaining transmission of nerve impulses [22]. The neurotransmitters acetylcholine (ACh) and butyrylcholine (BCh) are hydrolyzed with both cholinergic enzymes to provide choline (Ch) and acetate/butyrate, respectively. In addition, AChE hydrolyzes a variety of ChE species throughout the body of an individual, particularly in the liver, pancreas, central nervous system (CNS), and blood serum [23]. It exists in peripheral tissues, cholinergic and non-cholinergic fibers, erythrocyte membranes, neurons, muscles, and CNS. BChE, on the other hand, primarily links brain cells’ glial and endothelial cells [24]. By blocking the cholinesterase enzyme from degrading neurotransmitter ACh, AChE inhibitors boost the neurotransmitter’s impact and endurance [25]. Patients with neurodegenerative diseases who receive the standard pharmacological inhibitor treatments also have gastrointestinal issues, hepatotoxicity, nausea, and diarrhea. The most popular AChE and BChE inhibitors are tacrine (Figure 2B), rivastigmine, galantamine, donepezil, and corydaline. The tacrine medicines that are most frequently used for therapeutic purposes have several undesirable side effects, such as chills, skin rashes, nausea, vomiting, agitation, weight loss, stomach distress, and nausea [26]. In light of this, there is an increasing demand to produce and use new AGIs and ChEIs with established natural antioxidant characteristics [27]. However, there is little information about BChE’s physiological function. Thus, the development of medications to treat prevalent neurodegenerative diseases relied heavily on specific inhibitors of both cholinergic enzymes. They are well recognized as crucial to the management of AD [28].

The reversible hydration of carbon dioxide (CO_2_) to proton (H^+^) and bicarbonate (HCO_3_^−^) is catalyzed by carbonic anhydrases (CAs) metalloenzymes that include zinc ions (Zn^2+^) [29]. Acetazolamide is regularly used as inhibitors for CA isoenzymes (Figure 2C), however, acetazolamide, methazolamide, ethoxzolamide, dichlorphenamide, dorzolamide, and brinzolamide are also frequently used for this purpose. The primary cause of permanent loss of vision and the second-leading factor in blindness globally is glaucoma. High intraocular pressure (IOP), which eventually leads to permanent peripheral vision loss and blindness, is the hallmark of the ophthalmological condition known as glaucoma. [30]. The most characteristic feature of glaucoma is damage to the optic nerves and gradual deterioration of retinal ganglion cells, which are essential components of vision loss [31]. Additionally, glaucoma is the primary cause of blindness worldwide. It has also been predicted that the number of glaucoma patients worldwide will increase to 112 million by 2040 [32]. The best glaucoma therapy currently available consists of IOP reduction with medications, laser surgery, and traditional surgery. However, these treatments still have problems and limitations, such as drug side effects [33]. In particular, clinically utilized CAIs might lead to diarrhea, tinnitus, nausea, vomiting, and loss of appetite, taste, and smell. They may also cause numbness in the mouth and lips, tingling in the fingers and hands, nausea, and vomiting [34]. Hence, it is preferable for the inhibitor to have topical action to prevent the aforementioned adverse effects of CA inhibitors.

In this study, the antioxidant properties of baicalin hydrate were evaluated using some bioassays, such as Fe^3+^ reducing, Cu^2+^ reducing, and ABTS^•+^, DMPD^•+^, and DPPH^•^ scavenging effects. Another aim of this study is to compare the possible inhibition effects of baicalin hydrate on α-glycosidase, AChE and BChE, and CA II enzymes, which are associated with some common global diseases.

## 2. Materials and Methods

### 2.1. Chemicals

Sigma-Aldrich GmbH (Steinheim, Germany) provided the commercially available baicalin hydrate, α-tocopherol (99%), butylated hydroxyanisole (BHA, 99%), butylated hydroxytoluene (BHT, ≥99%), Trolox (97%), 1,1-diphenyl-2-picrylhydrazyl (DPPH), 2,2′-azino-bis(3-ethylbenzothiazoline-6-sulfonic acid) (ABTS), N,N-dimethyl-p-phenylenediamine (DMPD), and other chemicals.

### 2.2. Fe^3+^ Reducing Ability Assays

According to prior investigation [35], baicalin hydrate’s ability to reduce Fe^3+^ was achieved. Also, according to previous works, the Fe^3+^-reducing effect of baicalin hydrate was tested. Briefly, 0.75 mL of deionized water and different doses of baicalin hydrate (10–30 μg/mL) were combined with 1.25 mL sodium phosphate buffer (pH 6.6, 0.2 M) and 1.25 mL of K_3_[Fe(CN)_6_] (1%). After 30 min incubation at 30 °C, the reaction medium was acidified with 1 mL trichloroacetic acid (10%, TCA) and re-incubated in the dark for 30 min. Finally, 0.5 mL of FeCl_3_ (0.1%) was transferred to the solution. The absorbance values of baicalin hydrate and references were recorded at 700 nm. The increased absorbance of the reaction mixture reflects an increasing reduction ability [36].

### 2.3. Cu^2+^ Reducing Ability Assays

The technique of Apak et al. [37] was utilized to assess the cupric ions (Cu^2+^) reducing capacity of baicalin hydrate. After carrying out the required experimental procedures, the absorbances were detected at 450 nm using a spectrophotometer (Shimadzu, UV-1280, Kyoto, Japan). To do so, 0.2 mL CuCl_2_ solution (10 mM), 200 µL neocuproine solution (7.5 mM), and 200 µL CH_3_COONa buffer solution (1.0 M) were added to a test tube. The solution was mixed with different concentrations of baicalin hydrate (10–30 μg/mL). The total volume then reduced to 1.5 mL by adding distilled water and stored at room temperature. After 30 min incubation, the absorbances of the samples were measured at 450 nm. The increased absorbance of the reaction reflects increased reduction capacity.

### 2.4. DPPH^•^ Radical Scavenging Activities

The Blois technique [38] was used to test the DPPH^•^ scavenging capacity of baicalin hydrate [39]. Briefly, 0.25 mL of different concentrations of baicalin hydrate in ethanol (10–30 μg/mL) and 0.25 mL of DPPH radical solution (0.3 mM) in ethanol were combined with 1 mL ethanol in test tubes. The mixture was then incubated at 37 °C for 30 min. The DPPH radical scavenging activities of baicalin hydrate were measured at 517 nm. The increased absorbance of the reaction exhibits increased scavenging capability.

### 2.5. ABTS^•+^ Radical Scavenging Activities

ABTS^•+^ scavenging capacity of baicalin hydrate was measured according to a different work and was detected at 734 nm [40]. ABTS^•+^ was produced by the reaction of 2 mM ABTS in water with 2.45 mM potassium persulfate (K_2_S_2_O_8_), incubated in the dark at room temperature for five hours. Before usage, the ABTS^•+^ solution was diluted to obtain an absorbance of 0.750 ± 0.025 at 734 nm with phosphate buffer (0.1 M, pH 7.4). Then, 1 mL of ABTS^•+^ solution was added to 3 mL of baicalin hydrate in ethanol at different concentrations (10–30 µg/mL). After 30 min, the percentage inhibition of ABTS at 734 nm was calculated for each concentration relative to a blank absorbance.

### 2.6. DMPD^•+^ Radical Scavenging Activities

Baicalin hydrate was evaluated for its capacity to remove DMPD^•+^ using the Fogliano technique [41]. For this purpose, 0.5 mL of FeCl_3_ (50 mM) and 1.5 mL of DMPD solution were added to the buffer solution (pH 5.3, 0.1 M). Then, this mixture was added to 1 mL of different concentrations (10–30 μg/mL) of baicalin hydrate. Then, the total volume was adjusted to 4 mL using deionized water. Finally, 1 mL of DMPD radicals were added, and their absorbances were recorded at 505 nm.

### 2.7. Acetylcholinesterase/Butyrylcholinesterase Enzymes Inhibition Assay

The inhibition effects of baicalin hydrate on acetylcholinesterase (AChE) or butyrylcholinesterase (BChE) enzymes were realized according to Ellman’s process [42]. AChE was obtained from electric eel (*Electrophorus electricus*). BChE was purified from equine serum. The substrates of acetylthiocholine iodide/butyrylcholine iodide (AChI/BChI) were used for both cholinergic reactions. Briefly, 100 μL of Tris/HCl buffer (1.0 M, pH 8.0) and different baicalin hydrate concentrations were transferred to 50 μL of AChE/BChE solutions (5.32 × 10^−3^ EU) and incubated at 20 °C for 15 min. Then, 50 μL 5,5′-dithio-bis(2-nitro-benzoic) acid (DTNB, 0.5 mM,) and AChI/BChI were transferred to the mixture. The enzymatic reactions were initiated, and AChE/BChE activities were spectrophotometrically measured at 412 nm. One AChE/BChE enzyme unit is the quantity of enzymes that hydrolyze 1.0 mol of AChI/BChI to choline and acetate/butyrate per minute at pH 8.0 at 37 °C.

### 2.8. α-Glycosidase Enzyme Inhibition Assay

The inhibition effects of baicalin hydrate on α-glycosidase (from *Saccharomyces cerevisiae*) were assessed according to the procedure outlined by Tao et al. [43]. p-Nitrophenyl-D-glucopyranoside (p-NPG) was used as a substrate for α-glycosidase. Briefly, 75 μL phosphate buffer (pH 7.4) was mixed with 5 μL of the sample and 20 μL α-glycosidase enzyme solution in a phosphate buffer (0.15 U/mL, pH 7.4). After preincubation, 50 μL of p-nitrophenyl-D-glycopyranoside (p-NPG) in a phosphate buffer (5 mM, pH 7.4) was transferred, and the solution was incubated at 37 °C. The absorbance of the samples was measured at 405 nm. The amount of enzyme that catalyzed 1.0 mol of substrate per minute (pH 7.4) was one α-glycosidase unit.

### 2.9. Human Carbonic Anhydrase II Isoenzyme Inhibition Assay

hCA II isoenzyme was purified from fresh human erythrocytes. The erythrocytes were spun at 10,000× g for 30 min. The serum was then separated, and the pH was adjusted to 8.7 using solid Tris [44]. Sepharose-4B-L-Tirozyne sulfanilamide affinity column chromatography was used to purify the CA II isoenzyme [45]. The specimen was placed on the affinity column, and Tris-Na_2_SO_4_/HCl (22 mM/25 mM, pH: 8.7) was used to equilibrate it. Sodium acetate/NaClO_4_ (0.5 M, pH 5.6, 25 °C) was then used to elute the hCA II isozyme. The Bradford technique [46] was used to measure the protein quantity throughout the purification process. Bovine serum albumin was employed as a reference protein. SDS-PAGE was used to monitor the purity of the hCA II isoform [47]. Esterase activity was performed throughout hCA II’s purification and inhibition processes. The shift in absorbance at 348 nm was used to calculate the CA II isoenzyme activity [48]. One hCA II isoenzyme unit is the amount of hCA II, which had absorbance difference at 348 nm over a 3 min at 25 °C.

### 2.10. IC_50_ Values Determination

The IC_50_ values were determined using plots of activity (%) versus baicalin hydrate [49]. K_i_ values and other inhibition factors were determined using Lineweaver–Burk graphs [50].

### 2.11. Statistical Analysis

The experiments were repeated three times. The measurements are shown as mean ± SD. Tukey post hoc test was used after the one-way ANOVA; variations were assessed and considered significant when *p* < 0.05. The data are shown as the as mean ± SD of the average of three parallel observations (*n* = 3). When there are very significant differences between parameters in each group and the control value, the difference is shown with superscript a (*p* < 0.01). Significant deviations between each group’s parameter values and the control value are shown by superscript b (*p* < 0.5)

## 3. Results

**Figure 3:**
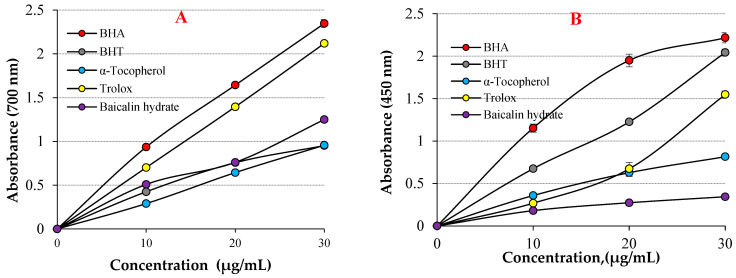
(**A**) Fe^3+^ reducing abilities of baicalin hydrate and standards, (**B**) Cu^2+^ reducing ability of baicalin hydrate and standards.

As shown in Table 1 and Figure 3A, baicalin hydrate showed excellent Fe^3+^ reducing ability, which was statistically proven to be highly significant (*p* < 0.01). The reducing ability of baicalin hydrate and standard antioxidants increased with increasing concentrations. For comparative purposes, the reference antioxidants BHA, BHT, α-tocopherol, and Trolox were employed. Stock solutions of all standards were prepared in ethyl alcohol. These standards were used because the antioxidant molecules BHA and BHT are strong, synthetic, and standard antioxidants; meanwhile, α-tocopherol and trolox, water-soluble analogues of tocopherol, were used as natural and standard antioxidants [51,52]. The Fe^3+^ reducing capacity of baicalin hydrate and the references were recorded in the following order: BHA (λ_700_: 2.347 ± 0.046, r^2^: 0.9086) >Trolox (λ_700_: 2.119 ± 0.001, r^2^: 0.9586) > baicalin hydrate (λ_700_: 1.249 ± 0.014, r^2^: 0.9848) > α-Tocopherol (λ_700_: 0.957 ± 0.018, r^2^: 0.9863) ≥ BHT (λ_700_: 0.952 ± 0.023, r^2^: 0.9154) at 30 μg/mL. The findings demonstrated that baicalin hydrate’s Fe^3+^ reducing capacity is lower than that of BHA and Trolox but comparable to that of BHT and α-Tocopherol. When examining the literature on this topic, it is observed that the Fe^3+^ reducing absorbance for usnic acid has been determined as 0.278 (r^2^: 0.9567) [53], 2.769 for caffeic acid (r^2^: 0.9945) [54], 0.432 (r^2^: 0.9981) for uric acid [55], 0.739 (r^2^: 0.9478) for coumestrol [16], 2.428 (r^2^: 0.9474) for tannic acid [56], 1.012 (r^2^: 0.9523) for spiraeoside [57], 0.967 (r^2^: 0.9938) for magnofluorine [28] and 2.509 (r^2^: 0.9906) for caffeic acid phenethyl ester [58] at similar concentration.

**Figure 4:**
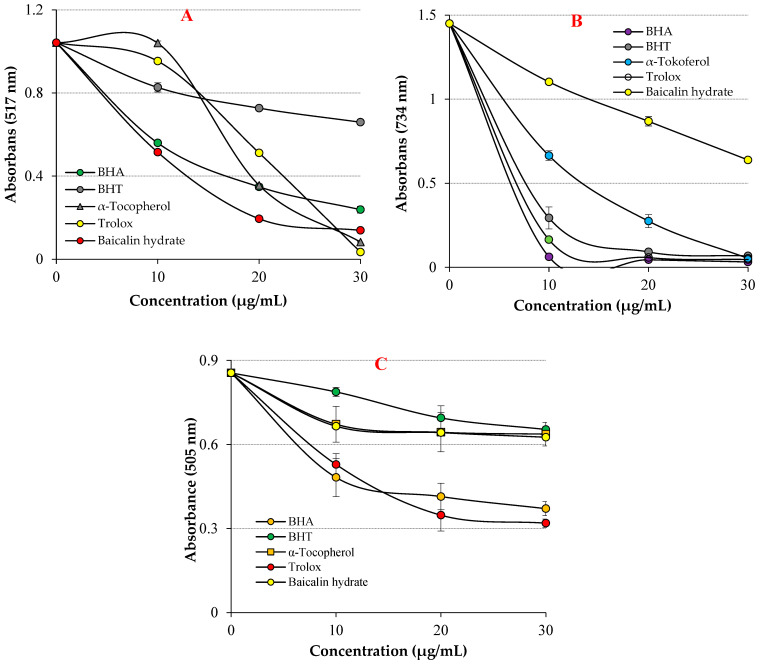
Radical scavenging abilities of baicalin hydrate and standard antioxidants, (**A**) DPPH^•^ scavenging, (**B**) ABTS^•+^ scavenging, (**C**) DMPD^•+^ scavenging effects.

In the DPPH free radical scavenging assay, the IC_50_ value for baicalin hydrate was 13.40 μg/mL (r^2^: 0.9940) (Table 2 and Figure 4A). Contrarily, the IC_50_ levels were 7.059 μg/mL for Trolox (r^2^: 0.9614), 10.10 μg/mL for BHA (r^2^: 0.9015), 11.31 μg/mL for α-tocopherol (r^2^: 0.9642) and 25.95 μg/mL for BHT (r^2^: 0.9221). The findings exhibited that baicalin hydrate had better DPPH radical scavenging activity in comparison to reference BHT and this scavenging ability comparable near to α-tocopherol, Trolox and BHA scavenging levels. The capacity of the compounds to scavenge free radicals is significantly influenced by the hydroxyl groups in both of their aromatic rings, according to the existing literature [59]. These findings exhibited that baicalin hydrate has more efficient DPPH free radical scavenging capacity when compared findings in previous studies.

Baicalin hydrate’s IC_50_ value for the ABTS^•+^ free radical scavenging test was 38.37 µg/mL (r^2^: 0.9888) (Table 2 and Figure 4B). In contrast, the IC_50_ levels were determined to be 6.16 μg/mL for Trolox (r^2^: 0.9692), 5.07 μg/mL for BHA (r^2^: 0.9356), 8.37 μg/mL for α-tocopherol (r^2^: 0.9015), and 6.99 μg/mL for BHT (r^2^: 0.9350). The findings showed that baicalin hydrate had efficient ABTS^•+^ radical scavenging activity compared to references.

DMPD radical was produced in situ by oxidizing with ferric chloride. The IC_50_ value for baicalin hydrate was determined to be 90.91 μg/mL (r^2^: 0.74.10) (Table 2 and Figure 4C). In contrast, the IC_50_ values were recorded as 4.33 μg/mL for Trolox (r^2^: 0.94.47), 11.99 μg/mL for BHA (r^2^: 0.9580), 7.11 μg/mL for α-tocopherol (r^2^: 0.9509), and 8.72 μg/mL for BHT (r^2^: 0.9375). The findings showed that baicalin hydrate had remarkable ABTS^•+^ radical scavenging activity compared to references.

**Figure 5:**
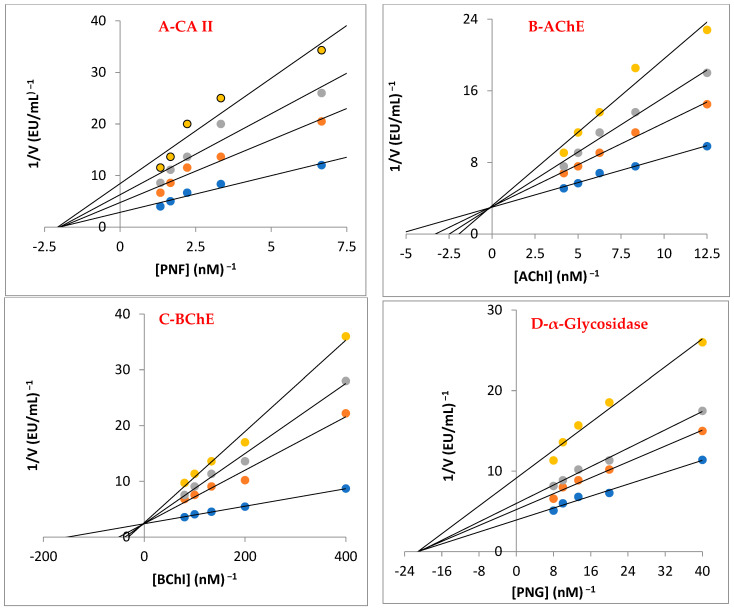
Lineweaver–Burk graphs for baicalin hydrate against carbonic anhydrase II (CA II) (**A**), acetylcholinesterase (AChE) (**B**), butyrylcholinesterase (BChE) (**C**), and α-glycosidase (**D**).

The cholinergic enzymes of AChE and BChE, which are targets for reducing AD symptoms, were also successfully inhibited by baicalin hydrate, with K_i_ values of 10.01 ± 2.86 and 3.50 ± 0.68 nM, respectively (Table 3, Figure 5B,C). Selectivity index (K_i_ for AChE/K_i_ BChE) for both cholinergic enzymes was 2.86. In this case, the baicalin hydrate had different affinities for both cholinergic enzymes. Additionally, Tacrine showed K_i_ values of 2.43 ± 0.92 nM for AChE (Figure 5B) and 5.99 ± 1.79 nM for BChE (Figure 5C). The most prevalent kind of dementia, AD, generally affects older adults. Chemical synapses and neuromuscular junctions are the key locations for AChE to act as the principal cholinesterase [60].

The metabolic disorder T2DM is characterized by hyperglycemia and inadequate endogenous insulin production or activity. High blood sugar levels are related to this metabolic illness. Recently, the research has concentrated on inhibiting α-glycosidase to regulate the breakdown of carbohydrates [43]. Baicalin hydrate had a K_i_ of 26.98 ± 9.91 nM towards α-glycosidase enzyme (Table 3 and Figure 5D). The findings clearly demonstrate that, when compared to acarbose (IC_50_: 22,800 nM), which is a typical α-glycosidase inhibitor, baicalin hydrate exhibited efficient α-glycosidase inhibition [61].

Phenolic substances block the CA isozymes due to the existence of functional groups in their scaffold that connect with the Zn^2+^ ions in the active-side cavity. With regard to the profiling experiment towards predominant and cytosolic hCA II isoenzyme, baicalin hydrate showed K_i_ value of 19.25 ± 1.79 nM (Table 3 and Figure 5A). In contrast, AZA, a therapeutic CA II inhibitor, exhibited a K_i_ value of 4.41 ± 0.35 nM against hCA II isoform. Numerous illnesses, including glaucoma, epilepsy, and oedema, are linked to physiologically dominant and cytosolic hCA II isoform, which is everywhere in cells [62].

## 4. Discussion

Phenolic compounds are the most common secondary metabolite in plants. These molecules have received a great deal of attention as possible herbal antioxidants since they are excellent radical scavengers and metal chelators. According to studies, the redox properties of phenol, both singlet oxygen quenchers and hydrogen donors, serve key functions in the compound’s antioxidant action. All plants contain phenolic compounds, which are a vital part of human nutrition and diet. They have sparked considerable attention due to their biological properties and antioxidant characteristics [63]. In spite of their redox characteristics, which allow them to operate as reducing agents, supply hydrogen ions, and sequester free radicals, polyphenolic substances exhibit antioxidant activity [64].

Antioxidants can function in a variety of ways by chelating metal ions, decomposing peroxides, removing ROS, and abstracting hydrogen. The reducing power, the most crucial characteristic of an antioxidant, is also reflected its electron-absorption capacity [65,66,67]. The findings definitely showed that baicalin hydrate administration reduced hepatic cytotoxicity and oxidative stress brought on by cadmium, and they give support for its therapeutic potential towards cadmium-induced oxidative stress [68]. In the current investigation, the antioxidant activity of baicalin hydrate was assessed using a variety of different techniques, comprising the removal of ABTS^•+^, DPPH^•+^, and DMPD^•+^, and reduction of potassium ferric cyanide complex and cupric ions (Cu^2+^). Considering their speed and accessible cost, a variety of in vitro antioxidant techniques and their variations are used to assess the antioxidant properties of pure substances. DMPD^•+^ scavenging activity, ABTS^•+^ scavenging activity, DPPH^•^ scavenging activity, potassium ferric cyanide reduction ability, and Cu^2+^ reducing activities are some of the most popular methods used to analyze the in vitro antioxidant compound activities.

The same concentration (30 μg/mL) of baicalin hydrate and the standards’ Cu^2+^ reduction capacities are shown in Table 1 and Figure 3B. The capacity to reduce Cu^2+^ and the various baicalin hydrate concentrations were shown to be positively correlated. Baicalin hydrate’s capacity to reduce Cu^2+^ was found to depend on increased concentrations (10–30 µg/mL). Cu^2+^ reducing ability of baicalin hydrate and the references at 30 μg/mL was identified according to: BHA (λ_450_: 2.216 ± 0.059, r^2^: 0.9928) > BHT (λ_450_: 2.044 ± 0.041, r^2^: 0.9937) > Trolox (λ_450_: 1.548 ± 0.024, r^2^: 0.9305) > α-Tocopherol (λ_450_: 0.816 ± 0.041, r^2^: 0.9897) ≥ baicalin hydrate (λ_450_: 0.344 ± 0.033, r^2^: 0.9517). The findings exhibited that Cu^2+^ reducing ability of baicalin hydrate is lower than those that used standard antioxidants. Furthermore, absorbance values for natural phenolic compound have been recorded in the previous literature, including usnic acid’s absorbance values for Cu^2+^ reduction as 0.277 (r^2^: 0.9836) [53], 0.468 (r^2^: 0.9729) for magnofluorine [28] 0.519 (r^2^: 0.9675) for spiraeoside [57] 0.762 for eugenol (r^2^: 0.9957) [69], 0.780 (r^2^: 0.9981) for coumestrol [16], 1.085 (r^2^: 0.8403) for resveratrol [70], 0.750 (r^2^: 0.9550) for taxifolin [71], and 1.314 (r^2^: 0.9682) for olivetol [72] at the same concentration.

In one related study, the addition of baicalin hydrate to the reduced product, then supplementing it with Fe^3+^ results in the formation of the Fe_4_[Fe(CN)_6_] complex, which has an absorbance of 700 nm [51]. Fe[(CN)_6_]^3+^ is reduced to Fe[(CN)_6_]^2+^ in the presence of reducing compounds [52].

The number and locations of hydroxyl groups (-OH) on polyphenol molecules, as well as the existence of double bonds or side chains, are only a few of the variables that affect the antioxidant capacity of these compounds. According to one theory, polyphenols containing more -OH groups have better antioxidant properties. To boost the antioxidant activities due to the low energy threshold for hydrogen transfer, the location of -OH groups and the presence of double bonds or side chains, such as ethylene, may reduce the enthalpy of dissociation of the O-H bond [59]. Multiple hydroxyl groups in the flavonoid molecules make them susceptible to ionization and oxidation in the aqueous phase. The physical and chemical properties of flavonoids, including their water solubility, stability, color, and adsorption characteristics, can be altered by the ionization and oxidation of -OH groups [73]. According to analysis of structure-activity linkage using preliminary assignment-score approach, phenolic hydroxyls and double bonds in rings A and B are enhancers, and the sugar moiety is an attenuator affecting antioxidant capacity [74]. Structurally, Baicalin’s aromatic rings have an ortho hydroxyl functional group (Figure 1). This structural element is responsible for baicalin’s divalent metal ion chelating and free radical scavenging properties [75]. A powerful antioxidant effect has been demonstrated for the baicalin hydrate, which has two pre-phenol hydroxyls and is capable of chelating iron and scavenging free radicals [76]. Because of the hydroxyl groups connected to aromatic rings in its structure, baicalin hydrate looks to be a potential antioxidant molecule. This phenolic may display antioxidant, reducing, and antiradical effects because of the aforementioned qualities. Baicalin hydrate contains -OH groups, which enhance its antioxidant capacity. The radical scavenging and chain-breaking abilities of phenolic hydrogen are enhanced by the ease with which it may be donated. The effectiveness of a molecule’s antioxidative defenses and capacity to scavenge free radicals generally increases with the amount of -OH groups it contains [77]. The unsubstituted -OH groups in baicalin hydrate showed antioxidant action.

Antioxidants’ capacity to scavenge free radicals and ROS was crucial in biological, pharmaceutical, and food applications as it helped to protect the goods and the body from harm [78]. The chromophore ABTS^•+^, DPPH^•^, and DMPD^•+^ scavenging tests employed are quick, easy to use, selective, affordable, and repeatable. To ascertain the antioxidant activities of pure compounds, it is rather typical to employ these radical scavenging activities. It is simple to employ the high detection sensitivity of the violet DPPH^•^, pink DMPD^•+^, and green-blue ABTS^•+^ radicals [66]. It is established that the quantity and location of -OH groups inside a molecule affect a phenolic’s ability to serve as an antioxidant and generate biological activity. Polymeric polyphenols offer more antioxidant benefits than monomeric polyphenols as a result of these characteristics [79]. The structure of baicalin hydrate causes interference in the DPPH free radicals, according to the mechanism described between baicalin hydrate and DPPH [80].

The DPPH radical scavenging activity was shown to increase with baicalin concentration. With an IC_50_ value of 64.92 µM, this finding showed that baicalin can quench the DPPH radical [81]. In a different investigation, baicalin, ascorbic acid, and BHT were revealed to have the highest absorbances of the three samples, and baicalin hydrate had a higher Fe^3+^ reducing capacity than ascorbic acid and BHT. In this experiment, BHT had relatively low reducing capacity. The outcomes of DPPH tests were slightly different. The findings suggested that baicalin, ascorbic acid, and BHT have a notable capacity to donate electrons to reactive free radicals, converting them into more stable non-reactive species and stopping the free radical chain reaction. The median inhibitory concentrations (IC_50_) for baicalin hydrate, ascorbic acid, and BHT were reported to be 17.0, 19.6, and 21.7 μg/mL, respectively [82]. It is possible to compare the free radical scavenging potentials of baicalin hydrate to various phenolic antioxidants whose DPPH radical scavenging capabilities were measured [54]. According to previous reports, IC_50_ value was determined to be 49.50 μg/mL for usnic acid [53], 3.30 μg/mL for CAPE [54], 16.06 μg/mL for eugenol [69], 6.96 μg/mL for resveratrol [69], 77.00 μg/mL for taxifolin [71], 10.58 μg/mL for magnofluorine [28], 17.77 μg/mL for olivetol [72], 20.0 mg/mL for silymarin [83], 25.95 μg/mL for coumestrol [16], 28.51 μg/mL for spiraeoside [57], 30.6 μg/mL for L-Adrenaline [84], and 34.9 μg/mL for curcumin, which were the first phenolic compounds to be purified from a plant source [85]. The results of this investigation show that baicalin hydrate is capable of scavenging DPPH radicals. It is generally known that baicalin hydrate and DPPH interact, and that radicals vanish after taking an electron or a hydrogen atom from the baicalin hydrate to form DPPH-H. To our knowledge, the baicalin hydrate’s DPPH scavenging mechanism has not yet been documented. On the basis of its resonance structure stability, the most likely possibility is that it stabilizes the radicals produced on the phenolic groups in the baicalin hydrate. Likewise, the structure of a baicalin hydrate molecule can switch from the radical forms specified to the neutral form by transitioning to a diketonic structure if it scavenges two DPPH in this manner.

ABTS^•+^ scavenging ability determined the capacity of pure compounds to reduce colors by combining directly with the ABTS radical [86]. The findings showed that baicalin hydrate has a larger capacity for scavenging ABTS^•+^, but that this capacity is less than that of positive controls. The capacity of slurry, beverages, extracts, and isolated compounds to scavenge radicals is also often assessed using the ABTS^•+^ and DPPH^•^ methods [87]. Baicalin, wogonoside, luteolin, luteolin 7-O-glycoside, and verbascoside were among the antioxidants from *S. alpina* shoot cultures that were assessed using the ABTS in vitro tests. The free radical scavenging activity of shoot cultures, represented as EC_50_, was shown to vary from 28.27 to 118.56 µg/mL using the ABTS test [88]. Compared to other pure and natural compounds, 10.41 g/mL was determined to be the IC_50_ value for usnic acid [53], 9.80 μg/mL for CAPE [58], 7.84 μg/mL for eugenol [69], 7.48 μg/mL (r^2^: 0.9952) for spiraeoside [57], 6.96 μg/mL for resveratrol [70], 0.83 μg/mL for taxifolin [71], 1.94 μg/mL for olivetol [62,72], 8.62 mg/mL for silymarin [83,85], 6.93 μg/mL for L-Adrenaline [84], 12.24 μg/mL for coumestrol [16], 18.07 μg/mL for curcumin [85], and 27.61 μg/mL for magnofluorine [28]. Effective ABTS radical scavenging was demonstrated by these compounds.

In the test samples, antioxidant molecules easily scavenged DMPD radicals. The IC_50_ values for DMPD^•+^ scavenging activity were more effective in other existing studies. For example, the IC_50_ values were found to be 33.00 μg/mL for usnic acid [53], 26.70 μg/mL for CAPE [54], 12.81 μg/mL for coumestrol [16], 10.04 μg/mL for eugenol [59], 8.15 μg/mL for spiraeoside [57], 9.5 μg/mL for resveratrol [70], 173.25 μg/mL for taxifolin [71], 19.25 μg/mL for olivetol [72], 15.6 μg/mL for L-Adrenaline [84], 15.16 μg/mL for magnofluorine [28], and 34.5 μg/mL for curcumin [85].

Natural products have a wide range of potential as novel medication leads since they have a significant chemical diversity, whether as pure compounds or standardized extracts. Plants are the most prevalent and economical source for new medication development [88]. The availability of increasingly effective drug candidate molecules with minimal or no adverse effects from medicinal plants and plant-based medications nowadays sparks interest in diabetes therapy [89]. In prior research, baicalin worked by activating the galanin receptor 2-glycose transporter 4 signal pathways and protecting against metabolic dysfunction and insulin resistance [90]. Baicalin hydrate exhibited a comparable effective Ki value of 26.98 ± 9.91 nM compared to Acarbose, which acts as a starch blocker, according to the results of the α-glycosidase inhibition. Generally, it has recognized that one of the techniques to avoid clinical problems affecting the neurological system, heart, kidneys, eyes, and other bodily systems is to manage blood glucose levels by blocking the α-glycosidase enzyme in T2DM. Diabetes is the primary factor in the progression several medical disorders, including erectile dysfunction, strokes, cardiovascular disease, renal failure, visual impairment, blindness, lower limb amputations, and inadequate wound healing [91]. In the onset, progression, and development of diabetes and its complications, elevated oxidative stress is a crucial and frequent concern.

Cholinesterase inhibitors (ChEIs), such as donepezil (5–10 mg daily), rivastigmine (1.5–6 mg daily), glutamate (16–24 mg daily), and N-methyl-D-aspartate receptor antagonist: Memantine (5–20 mg daily), are currently the approved medications for the clinical treatment of AD. However, these medications can only temporarily improve symptoms and cannot stop or reverse the progression of AD. Their clinical application is constrained by low compliance and significant adverse effects from the medications [92]. For this reason, herb medicine may be a substantial source of new drugs. The active flavonoid component of *S. baicalensis* as used in Chinese herb medicine may have extensive pharmacological effects on AD. In this study, we also determined the IC_50_ values of baicalin hydrate on cholinergic enzymes. K_i_ inhibition values of baicalin hydrate were found to be 10.01 and 3.50 nM on AChE and BChE, respectively. Baicalin hydrate’s K_i_ value on BChE enzyme were better than Tacrine Ki value’s. With 1.24 × 10^−4^ mol/L for 50% inhibition of AChE activity (IC_50_), baicalin demonstrated a clear inhibitory efficacy in a different study. Baicalin hydrate have been utilized to restore and rebalance neurotransmitter ACh levels, as noted in a study in the introduction to this paper, which may lessen AChE activity [22]. Furthermore, when the relevant literature examined, the K_i_ values of some natural compounds towards AChE and inhibition were calculated as 0.239 for usnic acid [53], 0.518 for CAPE [93], 3.4 nM for spiraeoside [57], 5.13 μg/mL for olivetol [72], 16.70 nM for taxifolin [69], and 21.43 nM for coumestrol [16]; therefore, these natural products inhibit the buildup of free radicals and ROS.

By losing protons (H^+^) from their -OH groups, phenolics have a mild acidic pH and transform into extremely water-soluble phenolate anions. The effectiveness of phenols’ inhibitory profiles on CA isoenzymes is also well established [94]. Notably, the phenolic -OH, -C=O, -OCH_3_ and -COOH groups, which may coordinate to the Zn^2+^ ion on the active-side of the CA cavity, are present in their scaffold [95]. We studied the inhibition effect of baicalin hydrate on the CA II isoenzyme and showed that results were too efficient compared with the standard of acetazolamide. One study demonstrated baicalin decreased the pathological alterations that high intraocular pressure caused in the retinas of glaucoma-affected mice. In order to control PI3K/AKT signaling both in vitro and in vivo, baicalin inhibits the progression of glaucoma [96]. Baicalin hydrate has not been studied in CA II isozyme inhibition before; this area is still open for research. Our study may be a pioneer for future studies. In this study, there were very efficient results that can be developed and applied in pharmacology and medicine investigations.

## 5. Conclusions

The origin of the baicalin hydrate molecule is the *Scutellaria baicalensis* Georgi radix plant. Baicalin hydrate is a potent flavonoid molecule with a powerful antioxidant effect that is also found in many other plants. It has been used in Eastern medicine for many diseases for thousands of years. This is a thorough investigation of the baicalin hydrate molecule, about which little is known in terms of molecular and enzyme interaction utilization and outcomes in the literature. We mainly studied the effects of this organic molecule on degenerative diseases that are common all over the world by using enzymes that have an impact on controlling the diseases. It has been shown that baicalin hydrate, when compared to standard options, has considerable and efficient antioxidant potential employed by multiple in vitro techniques. It has also been exhibited that baicalin hydrate, which possesses a wide range of essential biological properties, is an active substance that eliminates ROS and free radicals by giving hydrogen atoms (H) or electrons (e-) to free radicals. The outcomes of this study also unambiguously demonstrate that baicalin hydrate, a natural phenolic antioxidant that is safer, may be employed in pharmaceutical and food applications to stop or postpone the onset of oxidation processes. As well as maintaining nutritional value, it can increase the shelf-life of foods and medicines. The results obtained from this study show that baicalin hydrate efficiently inhibits several metabolic enzymes, such as acetylcholinesterase, butyrylcholinesterase, carbonic anhydrase II, and α-glycosidase. These enzyme inhibition properties of baicalin hydrate contribute to its antidiabetic, anticholinergic, and antiglaucoma properties.

## Figures and Tables

**Table 1:** Fe^3+^ and Cu^2+^ reduction abilities of baicalin hydrate and standards at 30 μg/mL concentration.

Antioxidants	Fe^3+^ Reducing		Cu^2+^ Reducing	
	λ (700 nm)	r^2^	λ (450 nm)	r^2^
BHA	2.347	0.9086	2.216	0.9928
BHT	0.952	0.9154	2.044	0.9937
Trolox	2.119	0.9586	1.548	0.9305
α-Tocopherol	0.957	0.9863	0.816	0.9897
Baicalin hydrate	1.249	0.9848	0.344	0.9517

**Table 2:** IC_50_ (μg/mL) values for DPPH^•^, ABTS^•+^ and DMPD^•+^ scavenging activities of baicalin hydrate and standard antioxidants.

Antioxidants	DPPH• Scavenging		ABTS•^+^ Scavenging		DMPD•^+^ Scavenging	
	IC_50_	r^2^	IC_50_	r^2^	IC_50_	r^2^
BHA	10.10	0.9015	5.07	0.9356	11.99	0.9580
BHT	25.95	0.9221	6.99	0.9350	8.72	0.9375
Trolox	7.05	0.9614	6.16	0.9692	4.33	0.9447
α-Tocopherol	11.31	0.9642	8.37	0.9015	7.11	0.9509
Baicalin hydrate	13.40	0.9940	38.37	0.9888	90.91	0.7410

**Table 3:** The enzyme inhibition values of baicalin hydrate towards carbonic anhydrase isoenzyme II (CA II), acetylcholinesterase (AChE), butyrylcholinesterase (BChE), and α-glycosidase (α-Gly).

Compounds	IC_50_ (nM)								K_i_ (nM, *n*: 3)			
	CA II	r^2^	AChE	r^2^	BChE	r^2^	α-Gly	r^2^	CA II	AChE	BChE	α-Gly
Baicalin hydrate	31.13	0.9562	14.65	0.9758	12.31	0.9524	24.62	0.9524	19.25 ± 1.79	10.01 ± 2.86	3.50 ± 0.68	26.98 ± 9.91
Acetazolamide *	8.37	0.9825	-	-	-	-	-	-	4.41 ± 0.35	-	-	-
Tacrine **	-	-	5.97	0.9706	8.37	0.9846	-	-	-	2.43 ± 0.92	5.99 ± 1.79	-
Acarbose ***	-	-	-	-	-	-	22,800	-	-	-	-	-

* Acetazolamide is a reference inhibitor of carbonic anhydrase II isoenzyme (CA II). ** Tacrine is a reference inhibitor for AChE. *** Acarbose is a reference inhibitor for α-glycosidase [43].

## Data Availability

Data available in a publicly accessible repository.
